# Supplementary material for: Mitochondrial origins of the pressure to sleep
Source: Nature. 2025 Jul 16;645(8081):722–8. doi: 10.1038/s41586-025-09261-y (PMC12443607; doi:10.1038/s41586-025-09261-y)
Supplement: Supplementary file 1 — Supplementary Tables 1–5. [file 41586_2025_9261_MOESM1_ESM.pdf]

nature portfolio

https://doi.org/10.1038/s41586-025-09261-y

Supplementary information

Mitochondrial origins of the pressure to sleep

In the format provided by the authors and unedited

Supplementary Table 1. Statistical analyses of data in Figures 2–4.

Figure	Statistical test	Pairwise comparison	Test statistic	*P*
**2b**	Two-sided Mann-Whitney test	Rested vs. sleep-deprived	U = 141	<0.0001
**2c**	Two-sided Mann-Whitney test	Rested vs. sleep-deprived	U = 218	0.0003
**2d**	Two-sided paired *t-test*	Before vs. after	t_7 = 3.192	0.0152
**2e**	Two-sided unpaired *t-test*	*R23E10>iATPSnFr* vs. *R23E10>iATPSnFr; CsChrimson*	t_{16} = 3.054	0.0076
**2f**	One-way ANOVA			
	Genotype		$F_{4,332}$ = 8.486	<0.0001
	Holm-Šidák test	*R23E10 ∩ VGlut-GAL4>Ucp4A* vs. *R23E10 ∩ VGlut-GAL4*	t_{332} = 2.501	0.0381
	Holm-Šidák test	*R23E10 ∩ VGlut-GAL4>Ucp4A* vs. *Ucp4A*	t_{332} = 5.024	<0.0001
	Holm-Šidák test	*R23E10 ∩ VGlut-GAL4>Ucp4C* vs. *R23E10 ∩ VGlut-GAL4*	t_{332} = 2.366	0.0381
	Holm-Šidák test	*R23E10 ∩ VGlut-GAL4>Ucp4C* vs. *Ucp4C*	t_{332} = 2.407	0.0381
**2g**	Kruskal-Wallis ANOVA			
	Experimental group		H_4 = 16.75	0.0008
	Dunn's test	*R23E10 ∩ VGlut-GAL4>mito-dR* (+ ret) vs. *R23E10 ∩ VGlut-GAL4>mito-dR* (- ret.)	Z = 3.687	0.0007
	Dunn's test	*R23E10 ∩ VGlut-GAL4>mito-dR* (+ retinal) vs. *mito-dR* (+ retinal)	Z = 3.404	0.0020
	Dunn's test	*R23E10 ∩ VGlut-GAL4>mito-dR* (+ retinal) vs. *R23E10 ∩ VGlut-GAL4* (+ retinal)	Z = 2.447	0.0432
**2h**	Mixed-effects model			
	Time		$F_{(2.920,777.1)}$ = 385.3	<0.0001
	Δp photogeneration		$F_{(3,268)}$ = 9.604	<0.0001
	Time x Δp photogeneration		$F_{(69,6121)}$ = 23.02	<0.0001
**3b**	Number of mitochondria			
	One-way ANOVA			
	Effect of SD, recovery		$F_{(2,140)}$ = 12.65	<0.0001
	Holm-Šidák test	Rested vs. sleep-deprived	t_{140} = 4.533	<0.0001
	Holm-Šidák test	Rested vs. recovered	t_{140} = 1.429	0.1551
	Two-sided unpaired *t-test*	Rested + AOX vs. sleep-deprived + AOX	t_{32}=1.235	0.2257
	Two-sided unpaired *t-test*	Rested + TrpA1 vs. sleep-deprived + TrpA1	t_{34}=0.5197	0.6067
	Volume			
	Kruskal-Wallis ANOVA			
	Effect of SD, recovery		H_3 = 34.38	<0.0001
	Dunn's test	Rested vs. sleep-deprived	Z = 2.265	0.0470
	Dunn's test	Rested vs. recovered	Z = 2.488	0.0257
	Two-sided Mann-Whitney test	Rested + AOX vs. sleep-deprived + AOX	U = 140.5	0.8985
	Two-sided unpaired *t-test*	Rested + TrpA1 vs. sleep-deprived + TrpA1	t_{34}=1.926	0.0625
	Sphericity			
	Kruskal-Wallis ANOVA			
	Effect of SD, recovery		H_3 = 46.70	<0.0001
	Dunn's test	Rested vs. sleep-deprived	Z = 2.737	0.0124
	Dunn's test	Rested vs. recovered	Z = 2.803	0.0101
	Two-sided unpaired *t-test*	Rested + AOX vs. sleep-deprived + AOX	t_{32}=0.3990	0.6925
	Two-sided unpaired *t-test*	Rested + TrpA1 vs. sleep-deprived + TrpA1	t_{34}=0.3626	0.7191
	Branch length			
	Kruskal-Wallis ANOVA			
	Effect of SD, recovery		H_3 = 47.53	<0.0001
	Dunn's test	Rested vs. sleep-deprived	Z = 3.151	0.0033
	Dunn's test	Rested vs. recovered	Z = 2.430	0.0302
	Two-sided Mann-Whitney test	Rested + AOX vs. sleep-deprived + AOX	U = 124	0.4958
	Two-sided Mann-Whitney test	Rested + TrpA1 vs. sleep-deprived + TrpA1	U = 143	0.5628
**3c**	Two-sided Mann-Whitney test	Rested vs. sleep-deprived	U = 11182	<0.0001
**3d**	Two-sided Mann-Whitney test	Rested vs. sleep-deprived	U = 449.5	<0.0001
**3e**	Two-sided unpaired *t-test*	Rested vs. sleep-deprived	t_{71} = 2.643	0.0101
**4b**	Two-way repeated-measures ANOVA			
	Time		$F_{(235,23782)}$ = 8.376	<0.0001
	Genotype		$F_{(10.86,5495)}$ = 622.2	<0.0001
	Time x Genotype		$F_{(5,506)}$ = 21.27	<0.0001

**4c, left**	One-way ANOVA			
	Genotype (Effect of GTPases)		$F_{(9,789)} = 26.13$	<0.0001
	Holm-Šidák test	*R23E10 ∩ VGlut-GAL4>Drp1* vs. *R23E10 ∩ VGlut-GAL4*	$t_{789} = 2.340$	0.0332
	Holm-Šidák test	*R23E10 ∩ VGlut-GAL4>Drp1* vs. *Drp1*	$t_{789} = 9.126$	<0.0001
	Holm-Šidák test	*R23E10 ∩ VGlut-GAL4>Opa1*^{RNAi} vs. *R23E10 ∩ VGlut-GAL4*	$t_{789} = 6.508$	<0.0001
	Holm-Šidák test	*R23E10 ∩ VGlut-GAL4>Opa1*^{RNAi} vs. *Opa1*^{RNAi}	$t_{789} = 8.986$	<0.0001
	Holm-Šidák test	*R23E10 ∩ VGlut-GAL4>Marf,Opa1* vs. *R23E10 ∩ VGlut-GAL4*	$t_{789} = 3.809$	0.0008
	Holm-Šidák test	*R23E10 ∩ VGlut-GAL4>Marf,Opa1* vs. *Marf*	$t_{789} = 2.950$	0.0098
	Holm-Šidák test	*R23E10 ∩ VGlut-GAL4>Marf,Opa1* vs. *Opa1*	$t_{789} = 3.649$	0.0011
	Holm-Šidák test	*R23E10 ∩ VGlut-GAL4>Drp1*^{RNAi} vs. *R23E10 ∩ VGlut-GAL4*	$t_{789} = 4.741$	<0.0001
	Holm-Šidák test	*R23E10 ∩ VGlut-GAL4>Drp1*^{RNAi} vs. *Drp1*^{RNAi}	$t_{789} = 2.398$	0.0332
**4c, right**	Kruskal-Wallis ANOVA			
	Genotype (Effect of PA regulators)		$H_5 = 110.8$	<0.0001
	Dunn's test	*R23E10 ∩ VGlut-GAL4>zuc*^{RNAi} vs. *R23E10 ∩ VGlut-GAL4*	Z = 3.150	0.0065
	Dunn's test	*R23E10 ∩ VGlut-GAL4>zuc*^{RNAi} vs. *zuc*^{RNAi}	Z = 7.715	<0.0001
	Dunn's test	*R23E10 ∩ VGlut-GAL4>Miga*^{RNAi} vs. *R23E10 ∩ VGlut-GAL4*	Z = 2.810	0.0198
	Dunn's test	*R23E10 ∩ VGlut-GAL4>Miga*^{RNAi} vs. *Miga*^{RNAi}	Z = 6.723	<0.0001
**4d, left**	Two-way repeated-measures ANOVA			
	Time		$F_{(1.478,548.2)} = 2.497$	0.0992
	Genotype		$F_{(2,371)} = 8.207$	0.0003
	Time x Genotype		$F_{(94,17437)} = 6.900$	<0.0001
**4d, centre**	Two-way repeated-measures ANOVA			
	Time		$F_{(1.487,645.2)} = 3.527$	0.0433
	Genotype		$F_{(3,434)} = 12.28$	<0.0001
	Time x Genotype		$F_{(141,20398)} = 18.01$	<0.0001
**4d, right**	Kruskal-Wallis ANOVA			
	Genotype		$H_6 = 78.02$	<0.0001
	Dunn's test	*R23E10 ∩ VGlut-GAL4>Opa1*^{RNAi} vs. *R23E10 ∩ VGlut-GAL4*	Z = 2.815	0.0244
	Dunn's test	*R23E10 ∩ VGlut-GAL4>Opa1*^{RNAi} vs. *Opa1*^{RNAi}	Z = 4.696	<0.0001
	Dunn's test	*R23E10 ∩ VGlut-GAL4>Marf,Opa1* vs. *R23E10 ∩ VGlut-GAL4*	Z = 2.612	0.0450
	Dunn's test	*R23E10 ∩ VGlut-GAL4>Marf,Opa1* vs. *Marf*	Z = 7.079	<0.0001
	Dunn's test	*R23E10 ∩ VGlut-GAL4>Marf,Opa1* vs. *Opa1*	Z = 4.396	<0.0001
**4f**	Mixed-effects model			
	Time		$F_{1.856,\ 114.6} = 227.5$	<0.0001
	Genotype		$F_{2,\ 69} = 15.56$	<0.0001
	Time x Genotype		$F_{42,\ 1297} = 5.822$	<0.0001
**4g**	Kruskal-Wallis ANOVA			
	Genotype		$H_3 = 1.465$	0.4806
	Dunn's test	*R23E10>mCD8:GFP* vs. *R23E10>Drp1*	Z = 0.8508	>0.9999
	Dunn's test	*R23E10>mCD8:GFP* vs. *R23E10>Marf,Opa1*	Z = 1.159	0.7392
	Dunn's test	*R23E10>Drp1* vs. *R23E10>Marf,Opa1*	Z = 0.3438	>0.9999
**4h**	Contingency table			
	χ2 test		$\chi^2_2 = 7.451$	0.0241
	Standardized residuals	*R23E10>mCD8:GFP* non-bursting	+ 0.62	
		R23E10>mCD8:GFP bursting	- 1.16	
		R23E10>Drp1;mCD8:GFP non-bursting	+ 0.35	
		R23E10>Drp1;mCD8:GFP bursting	- 0.66	
		R23E10>Marf,Opa1;mCD8:GFP non-bursting	- 1.07	
		R23E10>Marf,Opa1;mCD8:GFP bursting	+ 2.01	

Supplementary Table 2. Statistical analyses of data in Extended Data Figures 4–10.

Figure (ED)	Statistical test	Pairwise comparison	Test statistic	*P*
**4b**	Two-sided unpaired *t-test*	Rested vs. sleep-deprived	t_{43} = 0.4407	0.6616
**4c**	Kruskal-Wallis ANOVA			
	Genotype		H_5 = 42.82	<0.0001
	Dunn's test	*R23E10>Ucp4A* vs. *R23E10*	Z = 2.923	0.0139
	Dunn's test	*R23E10>Ucp4A* vs. *Ucp4A*	Z = 3.685	0.0009
	Dunn's test	*R23E10>Ucp4C* vs. *R23E10*	Z = 3.852	0.0005
	Dunn's test	*R23E10>Ucp4C* vs. *Ucp4C*	Z = 5.217	<0.0001
**4d**	Two-way repeated-measures ANOVA			
	Time		$F_{(85.36,25439)}$ = 1.886	<0.0001
	Genotype		$F_{(1,298)}$ = 23.15	<0.0001
	Time x genotype		$F_{(291,86718)}$ = 2.507	<0.0001
**4e**	Kruskal-Wallis ANOVA			
	Experimental group		H_4 = 17.92	0.0005
	Dunn's test	*R23E10>mito-dR* (+ retinal) vs. *R23E10>mito-dR* (- retinal)	Z = 2.930	0.0102
	Dunn's test	*R23E10>mito-dR* (+ retinal) vs. *R23E10* (+ retinal)	Z = 2.600	0.0279
	Dunn's test	*R23E10>mito-dR* (+ retinal) vs. *mito-dR* (+ retinal)	Z = 4.002	0.0002
**4f**	Mixed-effects model			
	Time		$F_{3.346,502.9}$ = 267.9	<0.0001
	Δp photogeneration		$F_{3,152}$ = 13.79	<0.0001
	Time x Δp photogeneration		$F_{69, 3457}$ = 15.95	<0.0001
**5d**	Number of mitochondria			
	Two-sided unpaired *t-test*	*TH>TrpA;R23E10>mito-GFP* vs. *R23E10>mito-GFP*	t_{22} = 1.381	0.1812
	Volume			
	Two-sided unpaired *t-test*	*TH>TrpA;R23E10>mito-GFP* vs. *R23E10>mito-GFP*	t_{22} = 3.780	0.0010
	Sphericity			
	Two-sided unpaired *t-test*	*TH>TrpA;R23E10>mito-GFP* vs. *R23E10>mito-GFP*	t_{22} = 2.527	0.0192
	Branch length			
	Two-sided unpaired *t-test*	*TH>TrpA;R23E10>mito-GFP* vs. *R23E10>mito-GFP*	t_{22} = 1.317	0.2013
**5f**	Number of mitochondria			
	Kruskal-Wallis ANOVA			
	Effect of SD, recovery		H_3 = 49.88	<0.0001
	Dunn's test	Rested vs. sleep-deprived	Z = 0.0303	>0.9999
	Dunn's test	Rested vs. recovered	Z = 6.311	<0.0001
	Two-sided unpaired *t-test*	Rested + AOX vs. sleep-deprived + AOX	t_{35} = 2.008	0.0524
	Two-sided unpaired *t-test*	Rested + TrpA1 vs. sleep-deprived + TrpA1	t_{52} = 1.530	0.1322
	Volume			
	One-way ANOVA			
	Effect of SD, recovery		$F_{(2,98)}$ = 61.53	<0.0001
	Holm-Šidák test	Rested vs. sleep-deprived	t_{98} = 3.106	0.0025
	Holm-Šidák test	Rested vs. recovered	t_{98} = 8.152	<0.0001
	Two-sided unpaired *t-test*	Rested + AOX vs. sleep-deprived + AOX	t_{35} = 1.466	0.1515
	Two-sided Mann-Whitney test	Rested + TrpA1 vs. sleep-deprived + TrpA1	U = 240.5	0.3212
	Sphericity			
	One-way ANOVA			
	Effect of SD, recovery		$F_{(2,98)}$ = 37.53	<0.0001
	Holm-Šidák test	Rested vs. sleep-deprived	t_{98} = 3.952	0.0001
	Holm-Šidák test	Rested vs. recovered	t_{98} = 5.144	<0.0001
	Two-sided unpaired *t-test*	Rested + AOX vs. sleep-deprived + AOX	t_{35} = 1.353	0.1848
	Two-sided unpaired *t-test*	Rested + TrpA1 vs. sleep-deprived + TrpA1	t_{52} = 1.297	0.2002
	Branch length			
	One-way ANOVA			
	Effect of SD, recovery		$F_{(2,98)}$ = 40.23	<0.0001
	Holm-Šidák test	Rested vs. sleep-deprived	t_{98} = 2.067	0.0414
	Holm-Šidák test	Rested vs. recovered	t_{98} = 6.895	<0.0001
	Two-sided Mann-Whitney test	Rested + AOX vs. sleep-deprived + AOX	U = 136.5	0.3868
	Two-sided Mann-Whitney test	Rested + TrpA1 vs. sleep-deprived + TrpA1	U = 229.5	0.2282

**5h**	One-way ANOVA			
	Genotype		$F_{(3,188)} = 2.452$	0.0648
	Holm-Šidák test	*R23E10>SPLICS* vs. *R23E10>OMM-mCherry*	$t_{188} = 2.223$	0.0800
	Holm-Šidák test	*R23E10>SPLICS* vs. *R23E10>tdTomato-Sec61β*	$t_{188} = 0.1674$	0.8673
	Holm-Šidák test	*R23E10>SPLICS* vs. *R23E10>CD4-tdTomato*	$t_{188} = 1.081$	0.4829
**6b**	Number of mitochondria			
	Two-sided unpaired *t-test*	Rested vs. sleep-deprived	$t_{24} = 0.3794$	0.7077
	Volume			
	Two-sided unpaired *t-test*	Rested vs. sleep-deprived	$t_{24} = 0.2465$	0.8074
	Sphericity			
	Two-sided unpaired *t-test*	Rested vs. sleep-deprived	$t_{24} = 0.4596$	0.6500
	Branch length			
	Two-sided unpaired *t-test*	Rested vs. sleep-deprived	$t_{24} = 0.6332$	0.5326
**6d**	Number of mitochondria			
	Two-sided Mann-Whitney test	Rested vs. sleep-deprived	U = 65	0.2534
	Volume			
	Two-sided Mann-Whitney test	Rested vs. sleep-deprived	U = 83	0.7637
	Sphericity			
	Two-sided Mann-Whitney test	Rested vs. sleep-deprived	U = 62	0.1953
	Branch length			
	Two-sided unpaired *t-test*	Rested vs. sleep-deprived	$t_{27} = 0.3933$	0.6972
**7b**	Volume			
	One-way ANOVA			
	Genotype		$F_{(3,47)} = 9.995$	<0.0001
	Holm-Šidák test	*R23E10>mito-GFP;Drp1* vs. *R23E10>mito-GFP;Marf,Opa1*	$t_{47} = 2.137$	0.0480
	Holm-Šidák test	*R23E10>mito-GFP;Drp1* vs. *R23E10>mito-GFP;Drp1*^{RNAi}	$t_{47} = 2.327$	0.0480
	Holm-Šidák test	*R23E10>mito-GFP;Opa1*^{RNAi} vs. *R23E10>mito-GFP;Marf,Opa1*	$t_{47} = 4.528$	0.0002
	Holm-Šidák test	*R23E10>mito-GFP;Opa1*^{RNAi} vs. *R23E10>mito-GFP;Drp1*^{RNAi}	$t_{47} = 4.539$	0.0002
	Sphericity			
	One-way ANOVA			
	Genotype		$F_{(3,47)} = 5.476$	0.0026
	Holm-Šidák test	*R23E10>mito-GFP;Drp1* vs. *R23E10>mito-GFP;Marf,Opa1*	$t_{47} = 2.178$	0.0344
	Holm-Šidák test	*R23E10>mito-GFP;Drp1* vs. *R23E10>mito-GFP;Drp1*^{RNAi}	$t_{47} = 2.501$	0.0316
	Holm-Šidák test	*R23E10>mito-GFP;Opa1*^{RNAi} vs. *R23E10>mito-GFP;Marf,Opa1*	$t_{47} = 3.032$	0.0118
	Holm-Šidák test	*R23E10>mito-GFP;Opa1*^{RNAi} vs. *R23E10>mito-GFP;Drp1*^{RNAi}	$t_{47} = 3.311$	0.0071
	Branch length			
	One-way ANOVA			
	Genotype		$F_{(3,47)} = 7.148$	0.0005
	Holm-Šidák test	*R23E10>mito-GFP;Drp1* vs. *R23E10>mito-GFP;Marf,Opa1*	$t_{47} = 2.202$	0.0326
	Holm-Šidák test	*R23E10>mito-GFP;Drp1* vs. *R23E10>mito-GFP;Drp1*^{RNAi}	$t_{47} = 2.956$	0.0097
	Holm-Šidák test	*R23E10>mito-GFP;Opa1*^{RNAi} vs. *R23E10>mito-GFP;Marf,Opa1*	$t_{47} = 3.247$	0.0064
	Holm-Šidák test	*R23E10>mito-GFP;Opa1*^{RNAi} vs. *R23E10>mito-GFP;Drp1*^{RNAi}	$t_{47} = 4.001$	0.0009
**7c**	Volume			
	Kruskal-Wallis ANOVA			
	Genotype		$H_4 = 80.92$	<0.0001
	Dunn's test	*R23E10>mito-GFP;Drp1* vs. *R23E10>mito-GFP;Marf,Opa1*	Z = 3.166	0.0062
	Dunn's test	*R23E10>mito-GFP;Drp1* vs. *R23E10>mito-GFP;Drp1*^{RNAi}	Z = 7.026	<0.0001
	Dunn's test	*R23E10>mito-GFP;Opa1*^{RNAi} vs. *R23E10>mito-GFP;Marf,Opa1*	Z = 4.152	0.0001
	Dunn's test	*R23E10>mito-GFP;Opa1*^{RNAi} vs. *R23E10>mito-GFP;Drp1*^{RNAi}	Z = 7.903	<0.0001
	Sphericity			
	One-way ANOVA			
	Genotype		$F_{(3,120)} = 8.747$	<0.0001
	Holm-Šidák test	*R23E10>mito-GFP;Drp1* vs. *R23E10>mito-GFP;Marf,Opa1*	$t_{120} = 2.421$	0.0170
	Holm-Šidák test	*R23E10>mito-GFP;Drp1* vs. *R23E10>mito-GFP;Drp1*^{RNAi}	$t_{120} = 3.001$	0.0065
	Holm-Šidák test	*R23E10>mito-GFP;Opa1*^{RNAi} vs. *R23E10>mito-GFP;Marf,Opa1*	$t_{120} = 3.635$	0.0012
	Holm-Šidák test	*R23E10>mito-GFP;Opa1*^{RNAi} vs. *R23E10>mito-GFP;Drp1*^{RNAi}	$t_{120} = 4.512$	<0.0001
	Branch length			
	Kruskal-Wallis ANOVA			
	Genotype		$H_4 = 24.68$	<0.0001
	Dunn's test	*R23E10>mito-GFP;Drp1* vs. *R23E10>mito-GFP;Marf,Opa1*	Z = 1.890	0.2350
	Dunn's test	*R23E10>mito-GFP;Drp1* vs. *R23E10>mito-GFP;Drp1*^{RNAi}	Z = 2.553	0.0427
	Dunn's test	*R23E10>mito-GFP;Opa1*^{RNAi} vs. *R23E10>mito-GFP;Marf,Opa1*	Z = 3.491	0.0019
	Dunn's test	*R23E10>mito-GFP;Opa1*^{RNAi} vs. *R23E10>mito-GFP;Drp1*^{RNAi}	Z = 4.590	<0.0001
**7d**	Two-way ANOVA			
	Drp1		$F_{1,118} = 29.05$	<0.0001
	Sleep history		$F_{1,118} = 22.82$	<0.0001
	Drp1 x sleep history		$F_{1,118} = 2.576$	0.1112

**8a**	One-way ANOVA			
	Genotype (Effect of GTPases)		$F_{11,714}$ = 33.80	<0.0001
	Holm-Šidák test	*R23E10>Drp1* vs. *R23E10*	t_{714} = 3.170	0.0079
	Holm-Šidák test	*R23E10>Drp1* vs. *Drp1*	t_{714} = 4.332	0.0001
	Holm-Šidák test	*R23E10>Opa1*^{RNAi} vs. *R23E10*	t_{714} = 7.729	<0.0001
	Holm-Šidák test	*R23E10>Opa1*^{RNAi} vs. *Opa1*^{RNAi}	t_{714} = 6.219	<0.0001
	Holm-Šidák test	*R23E10>Marf* vs. *R23E10*	t_{714} = 0.1551	0.8768
	Holm-Šidák test	*R23E10>Marf* vs. *Marf*	t_{714} = 1.726	0.1622
	Holm-Šidák test	*R23E10>Opa1* vs. *R23E10*	t_{714} = 2.963	0.0115
	Holm-Šidák test	*R23E10>Opa1* vs. *Opa1*	t_{714} = 4.505	<0.0001
	Holm-Šidák test	*R23E10>Opa1,Marf* vs. *R23E10*	t_{714} = 6.398	<0.0001
	Holm-Šidák test	*R23E10>Opa1,Marf* vs. *Opa1*	t_{714} = 7.802	<0.0001
	Holm-Šidák test	*R23E10>Opa1,Marf* vs. *Marf*	t_{714} = 7.301	<0.0001
	Holm-Šidák test	*R23E10>Drp1*^{RNAi} vs. *R23E10*	t_{714} = 8.503	<0.0001
	Holm-Šidák test	*R23E10>Drp1*^{RNAi} vs. *Drp1*^{RNAi}	t_{714} = 2.990	0.0115
**8b**	Kruskal-Wallis ANOVA			
	Genotype		H_7 = 94.10	<0.0001
	Dunn's test	*R23E10>zuc*^{RNAi} vs. *R23E10*	Z = 2.729	0.0381
	Dunn's test	*R23E10>zuc*^{RNAi} vs. *zuc*^{RNAi}	Z = 4.610	<0.0001
	Dunn's test	*R23E10>Miga*$^{RNAi\ 110737KK}$ vs. *R23E10*	Z = 4.876	<0.0001
	Dunn's test	*R23E10>Miga*$^{RNAi\ 110737KK}$ vs. *Miga*$^{RNAi\ 110737KK}$	Z = 3.615	0.0018
	Dunn's test	*R23E10>Miga*^{RNAi} vs. *R23E10*	Z = 6.911	<0.0001
	Dunn's test	*R23E10>Miga*^{RNAi} vs. *Miga*$^{RNAi\ 5492GD}$	Z = 4.722	<0.0001
**8c**	Kruskal-Wallis ANOVA			
	Genotype		H_5 = 78.08	<0.0001
	Dunn's test	*R23E10>Drp1-HA* vs. *R23E10*	Z = 6.175	<0.0001
	Dunn's test	*R23E10>Drp1-HA* vs. *Drp1-HA*	Z = 2.793	0.0209
	Dunn's test	*R23E10>Drp1*$^{Feany\ lab}$ vs. *R23E10*	Z = 7.861	<0.0001
	Dunn's test	*R23E10>Drp1*$^{Feany\ lab}$ vs. *Drp1*$^{Feany\ lab}$	Z = 4.875	<0.0001
**8d**	One-way ANOVA			
	Genotype		$F_{12,759}$ = 29.05	<0.0001
	Holm-Šidák test	*R23E10>Opa1*^{shRNA330266SH} vs. *R23E10*	t_{759} = 2.583	0.0199
	Holm-Šidák test	*R23E10>Opa1*^{shRNA330266SH} vs. *Opa1*^{shRNA330266SH}	t_{759} = 4.532	<0.0001
	Holm-Šidák test	*R23E10>Opa1*$^{RNAi\ \#32358}$ vs. *R23E10*	t_{759} = 3.592	0.0014
	Holm-Šidák test	*R23E10>Opa1*$^{RNAi\ \#32358}$ vs. *UAS-Opa1*$^{RNAi\ \#32358}$	t_{759} = 7.650	<0.0001
	Holm-Šidák test	*R23E10>Opa1*$^{miRNA.CDS}$ vs. *R23E10*	t_{759} = 8.277	<0.0001
	Holm-Šidák test	*R23E10>Opa1*$^{miRNA.CDS}$ vs. *UAS-Opa1*$^{miRNA.CDS}$	t_{759} = 8.663	<0.0001
	Holm-Šidák test	*R23E10>smOpa1 (X)* vs. *R23E10*	t_{759} = 3.679	0.0013
	Holm-Šidák test	*R23E10>smOpa1 (X)* vs. *smOpa1 (X)*	t_{759} = 2.478	0.0199
	Holm-Šidák test	*R23E10>smOpa1 (II)* vs. *R23E10*	t_{759} = 7.777	<0.0001
	Holm-Šidák test	*R23E10>smOpa1 (II)* vs. *UAS-smOpa1 (II)*	t_{759} = 2.947	0.0099
	Holm-Šidák test	*R23E10>smOpa1 (III)* vs. *R23E10*	t_{759} = 7.495	<0.0001
	Holm-Šidák test	*R23E10>smOpa1 (III)* vs. *UAS-smOpa1 (III)*	t_{759} = 9.094	<0.0001
**8e**	One-way ANOVA			
	Genotype		$F_{10,643}$ = 11.60	<0.0001
	Holm-Šidák test	*R23E10>Marf*$^{RNAi\ 40478GD}$ vs. *R23E10*	t_{643} = 1.901	0.2117
	Holm-Šidák test	*R23E10>Marf*$^{RNAi\ 40478GD}$ vs. *Marf*$^{RNAi\ 40478GD}$	t_{643} = 2.642	0.0496
	Holm-Šidák test	*R23E10>Marf*$^{RNAi\ 105261KK}$ vs. *R23E10*	t_{643} = 0.1334	0.8939
	Holm-Šidák test	*R23E10>Marf*$^{RNAi\ 105261KK}$ vs. *Marf*$^{RNAi\ 105261KK}$	t_{643} = 2.310	0.1017
	Holm-Šidák test	*R23E10>Marf*$^{RNAi\ \#55189}$ vs. *R23E10*	t_{643} = 1.276	0.4397
	Holm-Šidák test	*R23E10>Marf*$^{RNAi\ \#55189}$ vs. *Marf*$^{RNAi\ \#55189}$	t_{643} = 5.295	<0.0001
	Holm-Šidák test	*R23E10>smMarf (II)* vs. *R23E10*	t_{643} = 4.144	0.0003
	Holm-Šidák test	*R23E10>smMarf (II)* vs. *smMarf (II)*	t_{643} = 6.863	<0.0001
	Holm-Šidák test	*R23E10>smMarf (III)* vs. *R23E10*	t_{643} = 2.733	0.0443
	Holm-Šidák test	*R23E10>smMarf (III)* vs. *smMarf (III)*	t_{643} = 1.356	0.4397
**8f, left**	Two-way repeated-measures ANOVA			
	Time		$F_{1.442,363.3}$ = 13.33	<0.0001
	Genotype		$F_{2,\ 252}$ = 4.247	0.0153
	Time x genotype		$F_{94,11844}$ = 6.622	<0.0001
**8f, centre**	Two-way repeated-measures ANOVA			
	Time		$F_{1.367,\ 307.5}$ = 357.9	<0.0001
	Genotype		$F_{3,\ 225}$ = 3.296	0.0213
	Time x genotype		$F_{213,\ 15975}$ = 6.160	<0.0001
**8f, right**	Kruskal-Wallis ANOVA			
	Genotype		H_6 = 37.30	<0.0001
	Dunn's test	*R23E10>Opa1*^{RNAi} *vs. R23E10*	Z = 3.545	0.0020
	Dunn's test	*R23E10>Opa1*^{RNAi} *vs. Opa1*^{RNAi}	Z = 3.272	0.0053
	Dunn's test	*R23E10>Opa1,Marf vs. R23E10*	Z = 2.900	0.0186
	Dunn's test	*R23E10>Opa1,Marf vs. Marf*	Z = 4.266	<0.0001
	Dunn's test	*R23E10>Opa1,Marf vs. Opa1*	Z = 3.484	0.0025
**8g**	Kruskal-Wallis ANOVA			
	Genotype		H_3 = 7.932	0.0189
	Dunn's test	*R23E10>Marf-FLAG* vs. *R23E10*	Z = 1.862	0.1252
	Dunn's test	*R23E10>Marf-FLAG* vs. *Marf-FLAG*	Z = 0.8203	0.8241
**9a, b**	Kruskal-Wallis ANOVA			
	Genotype		H_{12} = 193.4	<0.0001
	Dunn's test	*R23E10>Drp1* vs. *R23E10*	Z = 2.229	0.3354

	Dunn's test	*R23E10>Drp1* vs. *Drp1*	Z = 4.528	<0.0001
	Dunn's test	*R23E10>Opa1*^{RNAi} vs. *R23E10*	Z = 0.04653	>0.9999
	Dunn's test	*R23E10>Opa1*^{RNAi} vs. *Opa1*^{RNAi}	Z = 6.468	<0.0001
	Dunn's test	*R23E10>Marf* vs. *R23E10*	Z = 0.6387	>0.9999
	Dunn's test	*R23E10>Marf* vs. *Marf*	Z = 1.700	>0.9999
	Dunn's test	*R23E10>Opa1* vs. *R23E10*	Z = 6.977	<0.0001
	Dunn's test	*R23E10>Opa1* vs. *Opa1*	Z = 3.132	0.0226
	Dunn's test	*R23E10>Marf,Opa1* vs. *R23E10*	Z = 6.806	<0.0001
	Dunn's test	*R23E10>Marf,Opa1* vs. *Marf*	Z = 5.751	<0.0001
	Dunn's test	*R23E10>Marf,Opa1* vs. *Opa1*	Z = 2.983	0.0371
	Dunn's test	*R23E10>Drp1*^{RNAi} vs. *R23E10*	Z = 5.139	<0.0001
	Dunn's test	*R23E10>Drp1*^{RNAi} vs. *Drp1*^{RNAi}	Z = 3.213	0.0170
**9c**	Kruskal-Wallis ANOVA			
	Genotype		H_5 = 9.949	0.0413
	Dunn's test	*R23E10>Marf,tdTomato* vs. *R23E10>Marf*	Z = 0.08112	>0.9999
	Dunn's test	*R23E10>Marf,tdTomato* vs. *tdTomato*	Z = 1.796	0.2899
	Dunn's test	*R23E10>Marf,OMM-mCherry* vs. *R23E10>Marf*	Z = 2.202	0.1106
	Dunn's test	*R23E10>Marf,OMM-mCherry* vs. *OMM-mCherry*	Z = 1.898	0.2310
**10a**	One-way ANOVA			
	Genotype		$F_{8,510}$= 16.13	<0.0001
	Holm-Šidák test	*GH146>Drp1* vs. *GH146*	t_{510} = 2.385	0.0842
	Holm-Šidák test	*GH146>Drp1* vs. *Drp1*	t_{510} = 3.031	0.0153
	Holm-Šidák test	*GH146>Opa1*^{RNAi} vs. *GH146*	t_{510} = 1.393	0.2211
	Holm-Šidák test	*GH146>Opa1*^{RNAi} vs. *Opa1*^{RNAi}	t_{510} = 6.731	<0.0001
	Holm-Šidák test	*GH146>Opa1* vs. *GH146*	t_{510} = 5.143	<0.0001
	Holm-Šidák test	*GH146>Opa1* vs. *Opa1*	t_{510} = 1.755	0.2211
	Holm-Šidák test	*GH146>Drp1*^{RNAi} vs. *GH146*	t_{510} = 1.609	0.2211
	Holm-Šidák test	*GH146>Drp1*^{RNAi} vs. *Drp1*^{RNAi}	t_{510} = 2.162	0.1187
**10b**	One-way ANOVA			
	Genotype		$F_{8,564}$ = 19.13	<0.0001
	Holm-Šidák test	*OK107>Drp1* vs. *OK107*	t_{564} = 1.638	0.2757
	Holm-Šidák test	*OK107>Drp1* vs. *Drp1*	t_{564} = 2.632	0.0429
	Holm-Šidák test	*OK107>Opa1*^{RNAi} vs. *OK107*	t_{564} = 6.202	<0.0001
	Holm-Šidák test	*OK107>Opa1*^{RNAi} vs. *Opa1*^{RNAi}	t_{564} = 0.09364	0.9764
	Holm-Šidák test	*OK107>Opa1* vs. *OK107*	t_{564} = 3.307	0.0070
	Holm-Šidák test	*OK107>Opa1* vs. *Opa1*	t_{564} = 0.1937	0.9764
	Holm-Šidák test	*OK107>Drp1*^{RNAi} vs. *OK107*	t_{564} = 2.396	0.0660
	Holm-Šidák test	*OK107>Drp1*^{RNAi} vs. *Drp1*^{RNAi}	t_{564} = 2.797	0.0316

Supplementary Table 3. *Drosophila* strains.

Name	Genotype	Source or Identifier	Ref.
R23E10	w[1118]; P{y[+t7.7] w[+mC]=GMR23E10-GAL4}attP2	BDSC_49032	58
R23E10-DBD	w[1118]; P{y[+t7.7] w[+mC]=R23E10-GAL4.DBD}attP2/TM3, Sb[1]	BDSC_69269	60
VGlut-AD	w[*]; Mi{Trojan-p65AD.2}VGlut[MI04979-Tp65AD.2]/CyO	BDSC_82986	61
GH146	y[1] w[1118]; P{w[+mW.hs]=GawB}GH146	BDSC_30026	62
OK107	w[*]; P{w[+mW.hs]=GawB}OK107 ey[OK107]/In(4)ci[D], ci[D] pan[ciD] sv[spa-pol]	BDSC_854	63
TH-LexA	w[*]; P{w[+mC]=ple-nlsLexA::GAD}2/CyO	BDSC_99050	64
6xEGFP	20XUAS-6XGFP @VK00018	Stowers lab<br>(Montana State University)	65
mCD8::GFP	UAS-mCD8::GFP	Luo lab<br>(Stanford University)	66
mito-GFP	w[1118]; P{w[+mC]=UAS-mito-HA-GFP.AP}2/CyO	BDSC_8442	67, 68
RFP	UAS-RFP (II)	Miesenböck lab<br>(University of Oxford)	
tdTomato	w[*]; P{w[+mC]=UAS-tdTom.S}3	BDSC_36328	
OMM-mCherry	w[1118]; P{w[+mC]=UAS-mCherry.mito.OMM}3/TM6B, Tb[1]	BDSC_66533	69
tdTomato-Sec61β	w[1118]; PBac{y[+mDint2] w[+mC]=20XUAS-tdTomato-Sec61beta}VK00037	BDSC_64746	70
CD4-tdTomato	y[1] w[*]; P{w[+mC]=UAS-CD4-tdTom}7M1	BDSC_35841	71
iATPSnFR	w[*]; UAS-iATPSnFR$^{1.0}$/CyO;+	Clandinin lab<br>(Stanford University)	72, 73
ATeam	UAS-ATeam1.03NL	Imai lab<br>(Juntendo University)	74, 75
AOX	UAS-AOX.F24	Jacobs lab<br>(Tampere University)	76
mito-dR	UAS-mito-dR (III)	Imai lab<br>(Juntendo University)	16, 77
CsChrimson::<br>tdTomato	20XUAS-IVS-CsChrimson::tdTomato @VK00005	Jayaraman lab<br>(Janelia Research Campus)	78, 79
UAS-TrpA1	w[*]; P{y[+t7.7] w[+mC]=UAS-TrpA1(B).K}attP16	BDSC_26263	80
LexAop-TrpA1	lexAop-dTrpA1 (III)	Waddell lab<br>(University of Oxford)	81
SPLICS	w[1118]; P{y[+t7.7] w[+mC]=UAS-SPLICS.s}attP16	Whitworth lab<br>(University of Cambridge)	82, 83
Mito-QC	w[1118]; P{y[+t7.7] w[+mC]=UAS-mito-QC}attP16	BDSC_91640	84, 85
Ucp4A	M{UAS-Ucp4A.ORF.3xHA.GW}ZH-86Fb	FlyORF_F003142	86, 87
Ucp4C	M{UAS-Ucp4C.ORF.3xHA.GW}ZH-86Fb	FlyORF_F002667	86, 87
Drp1	w[*]; P{w[+mC]=UAS-Drp1.D}3	BDSC_51647	88
Drp1-HA	UAS-Drp1-HA (III)	Chung lab<br>(Seoul National University)	89
Drp1$^{\text{Feany lab}}$	UAS-Drp1 (II)	Feany lab<br>(Harvard Medical School)	90
Opa1	UAS-Opa1-FLAG	Chung lab<br>(Seoul National University)	91
Marf	y[1] w[*]; P{w[+mC]=UAS-Marf.HA.S}3/T(2;3)TSTL, CyO: TM6B, Tb[1]	BDSC_67157	91
Marf-FLAG	UAS-Marf-FLAG	Chung lab<br>(Seoul National University)	89
Drp1^{RNAi}	w[*]; P{w[+mC]=UAS-Drp1.RNAi.CDS.S}3/T(2;3)TSTL, CyO: TM6B, Tb[1]	BDSC_67160	91
Opa1^{RNAi}	P{KK105706}VIE-260B	VDRC_106290	92
Opa1$^{\text{shRNA 330266SH}}$	P{VSH330266}attP40	VDRC_330266	92, 93
Opa1$^{\text{RNAi \#32358}}$	y[1] sc[*] v[1] sev[21]; P{y[+t7.7] v[+t1.8]=TRiP.HMS00349}attP2	BDSC_32358	93
Opa1$^{\text{RNAi \#67159}}$	w[*]; Bl[1]/CyO; P{w[+mC]=UAS-Opa1.miRNA.CDS}3	BDSC_67159	88
Opa1$^{\text{sm(X)}}$	UAS-smOpa1 (X)	Guo lab<br>(UCLA)	88
Opa1$^{\text{sm (II)}}$	UAS-smOpa1 (II)	Guo lab<br>(UCLA)	88
Opa1$^{\text{sm (III)}}$	UAS-smOpa1 (III)	Guo lab<br>(UCLA)	88
Marf$^{\text{RNAi 40478GD}}$	w1118; P{GD11094}v40478	VDRC_40478	92
Marf$^{\text{RNAi 105261KK}}$	P{KK105681}VIE-260B	VDRC_105261	92
Marf$^{\text{RNAi \#55189}}$	y[1] sc[*] v[1] sev[21]; P{y[+t7.7]v[+t1.8]=TRiP.HMC03883}attP40	BDSC_55189	93
Marf$^{\text{sm (II)}}$	UAS-smMarf (II)	Guo lab<br>(UCLA)	88
Marf$^{\text{sm (III)}}$	UAS-smMarf (III)	Guo lab<br>(UCLA)	88
zuc^{RNAi}	y[1] sc[*] v[1] sev[21]; P{y[+t7.7] v[+t1.8]=TRiP.GL00111}attP2	BDSC_35227	93
Miga^{RNAi}	w1118; P{GD3283}v5492	VDRC_5492	92
Miga$^{\text{RNAi 102948KK}}$	P{KK102948}VIE-260B	VDRC_110737	92
Drp1^{FLAG}	y[1] w[*]; P{w[+mC]=FLAG-FlAsH-HA-Drp1}3, Ki[1]	BDSC_42208	94

Supplementary Table 4. Image acquisition settings for quantitative microscopy.

Figure	Fluorophore(s)	Excitation (nm) | laser intensity (%)	Emission (nm)	Microscope	Laser intensity (WLL)	Objective	Pinhole (a.u.)	Gain	Zoom	Area (µm x µm)	z-step size (µm)	Bidirectional scan	Scan speed	Averaging	Resolution
**2b; ED 7d**	iATPSnFR<br>RFP	488 20%<br>555 15%	498 - 544<br>565 - 663	Leica TCS SP5	70%	HCX IRAPO L 25×/0.95 NA water	3	200%	9	68.96 x 68.96	1	Yes	700 Hz	3	1024 x 1024
**2c; ED 4a**	ATeam (mCFP)<br>ATeam (mVenus)	445 7.5%	454 - 507<br>516 - 693	Zeiss LSM980 with Airyscan2	-	Plan-Apochromat 40×/1.3 NA oil	1	820, 790	4	53.03 x 53.03	1	Yes	12	2	635 x 635
**3a-b; ED 5a-b, d, g; ED 6a-b; ED 7a-b**	GFP	488 20%	500-550	Olympus IX83 P2ZF with Yokogawa CSU-W1 super-resolution SoRa spinning-disk module	-	Olympus UPlanSApo 60×/1.30 NA silicon oil	SoRa Disk	2	3.2 (with SD-MGCA)	69.3 x 69.3	0.3	No scan: Photometrics Prime BSI cMOS camera	No scan: 500 ms exposure	-	2048 x 2048
**3c**	GFP<br>Alexa Fluor 633	488 20%<br>631 20%	500 - 540<br>642 - 690	Leica TCS SP5	70%	HCX IRAPO L 25×/0.95 NA water	1.5	200%	9.5	65.33 x 65.33	0.29	Yes	700 Hz	2	1024 x 1024
**3d**	SPLICS$_s$ (GFP)	488 20%	500 - 540	Leica TCS SP5	70%	HCX PL APO 40×/1.30 NA oil	1	200%	4	96.97 x 96.97	0.3	Yes	200 Hz	2	1024 x 1024
**3e**	Mito-QC (GFP)<br>Mito-QC (mCherry)	488 25%<br>587 15%	503 - 538<br>605 - 650	Leica TCS SP5	70%	HCX PL APO 40×/1.30 NA oil	3	100%	9.86	39.34 x 39.34	0.5	Yes	700 Hz	3	1024 x 1024
**ED 4b**	iATPSnFR<br>RFP	488 20%<br>555 15%	498 - 544<br>565 - 663	Leica TCS SP5	70%	HCX IRAPO L 25×/0.95 NA water	3	200%	3	206.77 x 206.77	1	Yes	700 Hz	3	2048 x 2048
**ED 5a-b, e-f; ED 7c**	GFP	488 30%	500 - 540	Leica TCS SP5	70%	HCX IRAPO L 25×/0.95 NA water	0.75	200%	9.86	64.51 x 64.51	0.29	Yes	400 Hz	3	1024 x 1024
**ED 5a-b; ED 6c-d**	GFP	488 30%	500 - 540	Leica TCS SP5	70%	HCX IRAPO L 25×/0.95 NA water	0.75	200%	5.4	114.87 x 114.87	0.29	Yes	400 Hz	3	2048 x 2048

Supplementary Table 5. Analysis of mitochondria in volume electron micrographs (*Drosophila* hemibrain v1.2.1).

dFBNs nominated by *R23E10-GAL4*

Body_ID	Neuron type
1105955480	FB6A_L
422191200	FB6A_L
5813061177	FB6A_L
5813055834	FB6A_R
5813061495	FB6A_R
946308203	FB6A_R
5813069331	FB6C_a_L
946645313	FB6C_a_R
5813071027	FB6C_b_L
5813050747	FB6C_b_L
762222901	FB6C_b_L
5813057169	FB6C_b_R
5813019588	FB6C_b_R
422876942	FB6E_L
5813049824	FB6E_R
915964590	FB6G_L
329289084	FB6G_R
5813058367	FB6I_L
452029745	FB6I_R
327730878	FB6Z_L
979065964	FB6Z_R
5813058368	FB7A_L
5813071028	FB7A_L
916292026	FB7A_L
297183251	FB7A_R
327203386	FB7A_R
5813020735	FB7A_R
420842989	FB7K_L
916288456	FB7K_L
294800293	FB7K_R
5813081818	FB7K_R

Uniglomerular olfactory PNs

Body_ID	Neuron type
1006068474	DP1m_adPN_L
5813061392	DM1_lPN_L
851235543	DM4_adPN_L
5813054061	DM2_lPN_L
850898421	DM3_adPN_L
1578658131	VA6_adPN_L
5813049472	DC4_adPN_L
5813050013	DM2_lPN_L
913650068	VM7d_adPN_L
1703479954	DC2_adPN_L
913650065	VM7d_adPN_L
882615309	DM6_adPN_L
913649999	DM6_adPN_L
5813057542	VM7d_adPN_L
881933350	DM6_adPN_L
1484850301	DL1_adPN_L
1671076884	D_adPN_L
5812994744	D_adPN_L
1670079387	DL1_adPN_L
1547273567	VM5d_adPN_L
1693577325	VM5d_adPN_L
913650164	VM7v_adPN_L
1887971628	VM5v_adPN_L
820545761	VM7v_adPN_L
1856259388	VM5d_adPN_L
1380503545	DC2_adPN_L
1889335463	DC2_adPN_L
5901195610	DM5_lPN_L
1671102680	DL4_adPN_L
1700428338	DL5_adPN_L
1702806746	DA3_adPN_L
1702815630	DA3_adPN_L
