# Supplementary material for: Mitochondrial origins of the pressure to sleep
Source: Nature. 2025 Jul 16;645(8081):722–8. doi: 10.1038/s41586-025-09261-y (PMC12443607; doi:10.1038/s41586-025-09261-y)
Supplement: Supplementary file 2 — Reporting Summary [file 41586_2025_9261_MOESM2_ESM.pdf]

nature portfolio

Corresponding author(s): Gero Miesenböck

Last updated by author(s): May 27, 2025

Reporting Summary

Nature Portfolio wishes to improve the reproducibility of the work that we publish. This form provides structure for consistency and transparency in reporting. For further information on Nature Portfolio policies, see our Editorial Policies and the Editorial Policy Checklist.

Statistics

For all statistical analyses, confirm that the following items are present in the figure legend, table legend, main text, or Methods section.

n/a	Confirmed	
☐	☒	The exact sample size (*n*) for each experimental group/condition, given as a discrete number and unit of measurement
☐	☒	A statement on whether measurements were taken from distinct samples or whether the same sample was measured repeatedly
☐	☒	The statistical test(s) used AND whether they are one- or two-sided<br>*Only common tests should be described solely by name; describe more complex techniques in the Methods section.*
☒	☐	A description of all covariates tested
☐	☒	A description of any assumptions or corrections, such as tests of normality and adjustment for multiple comparisons
☐	☒	A full description of the statistical parameters including central tendency (e.g. means) or other basic estimates (e.g. regression coefficient) AND variation (e.g. standard deviation) or associated estimates of uncertainty (e.g. confidence intervals)
☐	☒	For null hypothesis testing, the test statistic (e.g. *F*, *t*, *r*) with confidence intervals, effect sizes, degrees of freedom and *P* value noted<br>*Give P values as exact values whenever suitable.*
☒	☐	For Bayesian analysis, information on the choice of priors and Markov chain Monte Carlo settings
☒	☐	For hierarchical and complex designs, identification of the appropriate level for tests and full reporting of outcomes
☒	☐	Estimates of effect sizes (e.g. Cohen's *d*, Pearson's *r*), indicating how they were calculated

Our web collection on statistics for biologists contains articles on many of the points above.

Software and code

Policy information about availability of computer code

Data collection

Sleep behaviour data were collected using the DAM system (Trikinetics) or a MWP Z2S unit (Zantiks) .
Two-photon imaging data were collected using ScanImage 5.4.0, running on MATLAB2015a.
Confocal imaging data were collected using the Leica LAS AF 2.7.3.9723 or ZEN blue 3.3.
Super-resolution imaging data were collected using the Olympus cellSens Dimension 4.3.1 platform.
Electrophysiology data were acquired with custom protocols in pCLAMP 11.2 (Molecular Devices).

Data analysis

ScRNA-seq data were aligned and annotated according to James Nemesh, McCarroll Lab drop-seq core computational protocol V2.0.0 and analysed in R using Seurat v4.1.
Light microscopy data were analysed using existing, adapted, or newly developed (semi-)automated routines in Fiji 2.14.0/1.54f, using the DeconvolutionLab2 2.1.2 and Mitochondria Analyzer 2.3.1 plugins where indicated.
Two-photon imaging data were analysed in MATLAB 2023a.
Electrophysiological data were analysed using version 3.0c of the NeuroMatic package in Igor Pro 8.04 (WaveMetrics)..
Sleep behaviour data were analysed with the Sleep and Circadian Analysis MATLAB Program (SCAMP v3) or a custom MATLAB script for video tracking.
Gene ontologies were computed in PANTHER v17 or the ViSEAGO 1.4.0 and topGO 2.42.0 packages.
Behavioural, imaging, and electrophysiological data were analysed in Prism 10 (GraphPad) and SPSS Statistics 29 (IBM).

For manuscripts utilizing custom algorithms or software that are central to the research but not yet described in published literature, software must be made available to editors and reviewers. We strongly encourage code deposition in a community repository (e.g. GitHub). See the Nature Portfolio guidelines for submitting code & software for further information.

Data

Policy information about availability of data

All manuscripts must include a data availability statement. This statement should provide the following information, where applicable:

- Accession codes, unique identifiers, or web links for publicly available datasets
- A description of any restrictions on data availability
- For clinical datasets or third party data, please ensure that the statement adheres to our policy

Single-cell transcriptomic data were aligned to the Drosophila melanogaster genome release BDGP6.22 and can be found in NCBI's Gene Expression Omnibus repository under accession number GSE256379. All other data generated and analysed in this study are included in the Source Data.

Research involving human participants, their data, or biological material

Policy information about studies with human participants or human data. See also policy information about sex, gender (identity/presentation), and sexual orientation and race, ethnicity and racism.

Reporting on sex and gender	n/a
Reporting on race, ethnicity, or other socially relevant groupings	n/a
Population characteristics	n/a
Recruitment	n/a
Ethics oversight	n/a

Note that full information on the approval of the study protocol must also be provided in the manuscript.

Field-specific reporting

Please select the one below that is the best fit for your research. If you are not sure, read the appropriate sections before making your selection.

☒ Life sciences ☐ Behavioural & social sciences ☐ Ecological, evolutionary & environmental sciences

For a reference copy of the document with all sections, see nature.com/documents/nr-reporting-summary-flat.pdf

Life sciences study design

All studies must disclose on these points even when the disclosure is negative.

Sample size	Sample sizes are provided in each figure and extended data figure or its legend.<br>Sample sizes in behavioural experiments were chosen to detect 2-h differences in daily sleep with a power of 0.8.<br>Sample sizes in electrophysiological experiments are based on precedent (Kempf et al., Nature 2019).
Data exclusions	In single-cell transcriptomics, genes detected in fewer than 3 cells were excluded, and only cells associated with 800–10,000 UMIs and 200–5,000 transcripts were analysed.<br>Immobile flies (< 2 beam breaks per 24 h) in standard sleep assays in the Trikinetics Drosophila Activity Monitor system were excluded from the analysis.<br>In sleep analyses in the Zantiks unit, immobile flies (>98% zero-speed bins during ≥2 consecutive hours until the end of the recording) were excluded, beginning with the hour preceding the onset of immobility.<br>Only flies losing >95% of baseline sleep were included in measurements of sleep rebound after deprivation.<br>Only anatomically intact specimens from live flies (at the point of dissection) were used for mitochondrial morphometry.<br>Only dFBNs firing more than one action potential in response to depolarizing current injections, with resting potentials <–30 mV and series resistances <50 MΩ, were characterized.
Replication	All behavioural and imaging experiments were run at least three times, on different days and with different batches of flies. Behavioural and morphometric analyses were replicated in independent series of experiments, using two different dFBN-targeting GAL4 drivers and two imaging methods .All replicates are included in figures and extended data figures; only Extended Data Fig. 9d shows representative examples.
Randomization	Female flies of a given genotype, as indicated in Methods and figure legends, were randomly selected for analysis. Controls and experimental groups were always tested in parallel and in randomized order.
Blinding	The investigators were blind to group allocation in imaging experiments but not otherwise. Measurements and analyses were automated.

Reporting for specific materials, systems and methods

We require information from authors about some types of materials, experimental systems and methods used in many studies. Here, indicate whether each material, system or method listed is relevant to your study. If you are not sure if a list item applies to your research, read the appropriate section before selecting a response.

Materials & experimental systems

n/a	Involved in the study	
☐	☒	Antibodies
☒	☐	Eukaryotic cell lines
☒	☐	Palaeontology and archaeology
☐	☒	Animals and other organisms
☒	☐	Clinical data
☒	☐	Dual use research of concern
☒	☐	Plants

Methods

n/a	Involved in the study	
☒	☐	ChIP-seq
☐	☒	Flow cytometry
☒	☐	MRI-based neuroimaging

Antibodies

Antibodies used	Primary: Mouse anti-DDK, (AB_2622345, clone OTI4C5, F-tag-01 – TA100011), OriGene (1:1000).<br>Secondary: Goat anti-Mouse Alexa 633, (AB_2535719), Thermo Fisher Scientific (1:500).
Validation	The primary antibody was validated by the manufacturer in mammalian cells transfected with DDK-tagged vectors in Western blots and by immunofluorescence microscopy. A previous publication of ours validated the antibody in Drosophila (Rorsman et al., Nature 2025).<br>The secondary antibody was validated by the manufacturer in mammalian cells stained with mouse monoclonal primary antibody and by us in Drosophila (Rorsman et al., Nature 2025).

Animals and other research organisms

Policy information about studies involving animals; ARRIVE guidelines recommended for reporting animal research, and Sex and Gender in Research

Laboratory animals	Transgenic Drosophila melanogaster strains; genotypes indicated in Methods and Supplementary Table 3. Females aged 2–6 days after eclosion, as indicated in Methods, were used in experiments.
Wild animals	No wild animals were used in this study.
Reporting on sex	Female flies were used in all experiments because of their larger body size.
Field-collected samples	No field-collected samples were used in this study.
Ethics oversight	No ethical approval was required for research on Drosophila melanogaster.

Note that full information on the approval of the study protocol must also be provided in the manuscript.

Plants

Seed stocks	*Report on the source of all seed stocks or other plant material used. If applicable, state the seed stock centre and catalogue number. If plant specimens were collected from the field, describe the collection location, date and sampling procedures.*
Novel plant genotypes	*Describe the methods by which all novel plant genotypes were produced. This includes those generated by transgenic approaches, gene editing, chemical/radiation-based mutagenesis and hybridization. For transgenic lines, describe the transformation method, the number of independent lines analyzed and the generation upon which experiments were performed. For gene-edited lines, describe the editor used, the endogenous sequence targeted for editing, the targeting guide RNA sequence (if applicable) and how the editor was applied.*
Authentication	*Describe any authentication procedures for each seed stock used or novel genotype generated. Describe any experiments used to assess the effect of a mutation and, where applicable, how potential secondary effects (e.g. second site T-DNA insertions, mosiacism, off-target gene editing) were examined.*

Flow Cytometry

Plots

Confirm that:

☒ The axis labels state the marker and fluorochrome used (e.g. CD4-FITC).

☒ The axis scales are clearly visible. Include numbers along axes only for bottom left plot of group (a 'group' is an analysis of identical markers).

☒ All plots are contour plots with outliers or pseudocolor plots.

☒ A numerical value for number of cells or percentage (with statistics) is provided.

Methodology

Sample preparation	Freshly dissected fly brains were dissociated enzymatically and mechanically and filtered as described in Methods. Dead cells were excluded with the help a DAPI viability dye (1 µg ml-1, BD Pharmingen).
Instrument	MoFlo Astrios (Beckman Coulter) or FACSAria III (Becton Dickinson)
Software	FACSDiva (Becton Dickinson)
Cell population abundance	Approximately 15 EGFP-positive cells per dissected brain at a concentration of 300 cells/µl. Purity was ascertained visually by fluorescence microscopy.
Gating strategy	Single cells were gated for based on forward and side scatter parameters of wild-type fly brains, followed by subsequent gating for EGFP-positive and EGFP-negative cells (Extended Data Fig. 1b).

☒ Tick this box to confirm that a figure exemplifying the gating strategy is provided in the Supplementary Information.
